# Biological Control of Tomato Bacterial Wilt, Kimchi Cabbage Soft Rot, and Red Pepper Bacterial Leaf Spot Using *Paenibacillus elgii* JCK-5075

**DOI:** 10.3389/fpls.2020.00775

**Published:** 2020-07-01

**Authors:** Khanh Duy Le, Jueun Kim, Nan Hee Yu, Bora Kim, Chul Won Lee, Jin-Cheol Kim

**Affiliations:** ^1^Department of Agricultural Chemistry, Institute of Environmentally Friendly Agriculture, College of Agriculture and Life Sciences, Chonnam National University, Gwangju, South Korea; ^2^Department of Chemistry, Chonnam National University, Gwangju, South Korea

**Keywords:** antibacterial activity, mode of action, *Paenibacillus*, pelgipeptins, plant pathogenic bacteria

## Abstract

The over and repeated use of chemical bactericides to control plant bacterial diseases has resulted in unwanted effects, such as environmental pollution, residual toxicity, and resistance buildup in bacterial pathogens. Many previous studies have aimed to develop biological control agents to replace chemical bactericides. In this study, the antibacterial efficacy of the fermentation broth of *Paenibacillus elgii* JCK-5075 and its antibacterial compounds were evaluated against plant pathogenic bacteria, using both *in vitro* and *in vivo* bioassays. Pelgipeptins (PGPs) A, B, C, and D that were isolated from *P. elgii* JCK-5075 displayed broad-spectrum antibacterial activity against various plant pathogenic bacteria. The fermentation broth of *P. elgii* JCK-5075, at 5-fold dilution, effectively suppressed the development of tomato bacterial wilt, Kimchi cabbage soft rot, and red pepper bacterial leaf spot in pot experiments with control values of 81, 84, and 67%, respectively. PGP-A and C, at 200 μg/ml, were also found to markedly reduce the development of Kimchi cabbage bacterial soft rot by 75% and tomato bacterial wilt by 83%, respectively, and their disease control efficacy was comparable to that of oxolinic acid with control values of 81 and 85%, respectively. Additionally, the antibacterial activity of PGP-C was found to be directly correlated with membrane damage mechanisms. These results indicates that *P. elgii* JCK-5075 producing PGPs could be used as a biocontrol agent for the control of plant bacterial diseases. This is the first report on the *in vitro* and *in vivo* antibacterial activity of PGPs against bacterial plant pathogens.

## Highlights

– We found that pelgipeptins isolated from *P. elgii* JCK-5075 showed very strong *in vitro* and *in vivo* antibacterial activity against plant pathogenic bacteria. Additionally, the antibacterial mechanism of pelgipeptin-C was elucidated to be membrance damage.

## Introduction

Even though fungal diseases are more numerous and severe in plants, there are also several important pathogenic bacteria that can cause significant annual losses on a global scale, specifically in moist and hot climatic conditions (Egli and Sturm, 1981). The 10 most important bacterial plant pathogens, based on their scientific and economic importance are *Pseudomonas syringae* pathovars, *Ralstonia solanacearum*, *Agrobacterium tumefaciens*, *Xanthomonas oryzae* pv. *oryzae*, *Xanthomonas campestris* pathovars, *Xanthomonas axonopodis* pathovars, *Erwinia amylovora*, *Xylella fastidiosa*, *Dickeya* (*dadantii* and *solani*), and *Pectobacterium carotovorum* (Mansfield et al., 2012). Bacterial diseases in plants, however, are difficult to control for the following reasons: (1) bacterial pathogens under optimal environmental conditions can quickly increase their population sizes; (2) some soil-borne bacteria are an absence of effective fumigants for soil-borne bacteria; and (3) many pathogenic bacteria predominantly colonize the internal spaces of plants, while many of the standard control measures only target the plant surface (Sundin et al., 2016).

Over the last few decades, chemical control strategies have been applied to control plant diseases. However, the over and repeated use of several chemical pesticides, such as antibiotics and copper compounds, has caused pollution of the soil environment, a decrease in the population of beneficial microorganisms, residual toxicity on food commodities, and the occurrence of bactericide-resistant strains. In order to replace chemical pesticides, biological control agents, including bioactive metabolites produced by microorganisms or the microbial cells themselves, have previously been intensively studied (Roth et al., 1997; Kim et al., 2009; Lugtenberg and Kamilova, 2009; Sundin et al., 2016; Tsuda et al., 2016). The interest in biological control agents has increased, owing to their benefits, which include that they may potentially be self-sustaining as they may spread on their own after initial establishment; that they may require a reduced input of non-renewable resources; and that they may result in long-term disease suppression in the environment. The biological agents that have been used previously were mostly based on bacteria (90%) and fungi (10%) (Yuliar et al., 2015).

Bacteria produce a wide array of compounds to suppress their competitors and colonize new habitats, giving them a competitive advantage. Bacteria capable of thriving in extreme conditions are very likely to evolve special adaptations and produce unique molecules or compounds that may have beneficial uses. The use of beneficial bacteria is considered to be one of the most promising methods for more rational and safe crop-management practices (Fravel, 2005). Bacterial control agents have previously predominantly been from the genera of *Pseudomonas*, *Bacillus*, *Streptomyces*, and *Paenibacillus* (Shoda, 2000). Recently, many papers have reported the biocontrol potential of *Paenibacillus* spp. (Beatty and Jensen, 2002; Kim et al., 2009; Grady et al., 2016; Kumar et al., 2016). *Paenibacillus* species are a Gram variable, rod-shaped, endospore forming, and facultative anaerobes that were reclassified from *Bacillus* in 1993 by an extensive comparative analysis of their 16S ribosomal RNA gene sequences (Ash et al., 1993; Grady et al., 2016). Like other bacteria, the biocontrol capabilities of *Paenibacillus* spp. come from the promotion of plant growth, the induction of the plant’s own resistance, competition for nutrient and space conditions, antibiosis, and parasitism (Chung et al., 2000; Hong and Meng, 2003; He et al., 2007; Antonopoulos et al., 2008; Algam et al., 2010; Phi et al., 2010; Lee et al., 2012; Nguyen et al., 2015; Zhou et al., 2016). *Paenibacillus* species can be used in combination with other chemical products, as they have the capability to tolerate commercial fungicides or insecticides (Singh et al., 2009). Members of *Paenibacillus* are known to produce non-ribosomal lipopeptides with potent antimicrobial activities, such as polymyxin B by *P. polymyxa*, battacin by *P. tianmuensis*, pelgipeptins by *P. ehimensis*, and *P. elgii* produced PGPs (Khan et al., 2008; Wu et al., 2010; Ding et al., 2011; Shaheen et al., 2011; Qian et al., 2012; Huang et al., 2013).

As for the disease control efficacy of *Paenibacillus* species, the *P. polymyxa* strain KNUC265 was reported to protect pepper and tobacco from *X. axonopodis* and *Erwinia carotovora* (*P. carotovorum*), respectively, using bacterial volatiles and diffusible metabolites as elicitors of induced systemic resistance (Phi et al., 2010). *P. polymyxa* E681 was also shown to protect *Arabidopsis thaliana* against *P. syringae* via induced systemic resistance (Lee et al., 2012). Octapeptins and paenibacterin, that are cyclic lipopeptides produced by *Paenibacillus*, are active against both gram-negative and gram-positive bacteria (Huang and Yousef, 2014; Cochrane and Vederas, 2016). Fusaricidins are known to be active against many important phytopathogens and a variety of gram-positive bacteria (Kajimura and Kaneda, 1996). Additionally, pelgipeptin (PGP)-E that was isolated from *P. elgii* BC34-6, was reported to be active against both gram-negative and gram-positive bacteria (Kim et al., 2018). Recently, our research group reported that PGP-A, B, C, and D, that were produced by *P. elgii* JCK1400 strain, showed strong antifungal activity against plant pathogenic fungi and PGP-C was effective at controlling tomato gray mold and wheat leaf rust (Kim et al., 2019). However, the information on the disease control efficacy of the antibacterial metabolites produced by the *Paenibacillus* species, against plant bacterial diseases and their antibacterial action mechanisms, was limited.

During the discovery of the antimicrobial metabolites, we found that the PGP-A, B, C, and D, that were isolated from the *P. elgii* JCK-5075, were also highly active at inhibiting the cell growth of various phytopathogenic bacteria. To the best of our knowledge, there is no previous report on the disease control efficacy of PGPs against bacterial plant diseases and their mode of action in phytopathogenic bacteria. Therefore, the objectives of this study were as follows: (1) to examine the *in vitro* antibacterial activity of the fermentation filtrate of *P. elgii* JCK-5075 and the purified PGPs against 14 plant pathogenic bacteria; (2) to evaluate the biocontrol efficacy of the fermentation broth of the JCK-5075 and the purified PGPs against bacterial plant diseases, and (3) to elucidate the mode of action of PGP-C on plant pathogenic bacteria.

## Materials and Methods

### Culture Conditions for JCK-5075

Several bacterial strains were isolated from a soil sample which was collected from one red pepper land, Daejeon city, South Korea in 2015. Among the strains, one strain (JCK-5075) showed very strong *in vitro* antibacterial activity against *R. solanacearum.* The bacterial strain JCK-5075 was cultured on tryptic soy agar (TSA; Becton, Dickinson and Co., Sparks, MD, United States) medium at 30°C for 3 days and then stored at −80°C in 30% glycerin until use.

### Identification of Antagonistic Bacteria

For the identification of JCK-5075, genomic DNA was extracted using the bacterial genomic DNA purification kit (ELPIS-Biotech, Daejeon, South Korea). The 16S rRNA gene was amplified by PCR using specific primer pair sets and sequencing (Genotech Co., Daejeon, South Korea). Cycles consisted of an initial denaturation at 95°C for 10 min, followed by 40 cycles (95°C for 30 s, 60°C for 30 s, 72°C for 40 s), and a final extension at 72°C for 10 min. The gene sequences obtained was compared with the database from GenBank using BioEdit version 5.0.9.1 (Kim et al., 2018). The phylogenetic trees were constructed by the neighbor-joining method in MEGA version 6.0 with 1000 bootstrap replicates.

### Plants

The seeds of tomato (cultivar “Seokwnag;” Farm Hannong Co., Ltd., Seoul, South Korea), Kimchi cabbage (cultivar “Chunkwang;” Sakada Korea, Seoul, South Korea), and red pepper (cultivar “Josaengsintopgochu;” Non-gwoobio Co., Ltd., Suwon, South Korea), were sown in vinyl pots (6.0 cm diameter) (1 seed per pot) containing nursery soil (Punong nursery series #5; Seoulbio Co., Ltd., Gyeongju, South Korea), and kept in an incubation room with 12 h of daylight per day. The plants were transplanted to larger plastic pots (7.5 cm diameter) 24 h prior to the treatments.

### Plant Pathogenic Bacteria

Fourteen pathogenic bacteria were used in this study: *Acidovorax avenae* subsp. *cattleyae* (bacterial brown spot of orchid), *Acidovorax konjaci* (bacterial black spot of cucumber), *A. tumefaciens* (crown gall of apple), *Burkholderia glumae* (bacterial panicle blight of rice), *P. carotovorum* subsp. *carotovorum* (bacterial soft rot of Kimchi cabbage), *Pectobacterium chrysanthemi* (bacterial soft rot of aloe), *P. syringae* pv. *actinidiae* (bacterial canker of kiwifruit), *P. syringae* pv. *lachrymans* (bacterial angular leaf spot of cucumber), *Xanthomonas euvesicatoria* (bacterial leaf spot of red pepper), *Xanthomonas arboricola* pv. *pruni* (bacterial spot of stone fruit), *X. axonopodis* pv. *citri* 24-20 (bacterial canker of citrus), *X. oryzae* pv. *oryzae* (bacterial blight of rice), *R. solanacearum* SL341 (bacterial wilt of tomato), and *Clavibacter michiganensis* subsp. *michiganensis* (bacterial canker of tomato). All plant pathogenic bacteria were isolated from infected plant tissues in South Korea by Dr. S. D. Lee of the National Academy of Agricultural Sciences, Prof. S. W. Lee of Dong-A University, and Prof. Y.-G. Ko of the Suncheon National University. The bacteria were all cultured on Tryptic Soy Agar (TSA) and Tryptic Soy Broth (TSB) medium at 30°C, except for *Xanthomonas* spp. which was incubated at 28°C.

### Purification and Structural Determination of PGPs

The purification and structural determination of the antimicrobial substances from the culture supernatant of the *P. elgii* JCK-5075 was conducted as previously reported (Kim et al., 2019). Briefly, cell-free supernatant was obtained by centrifugation of the culture broth. Then, the supernatant was loaded onto a Diaion HP-20 column (IONTEC, South Korea). The fractions were eluted using a stepwise ethanol gradient (0, 20, 40, 60, 80, and 100%, v/v). Each fraction was concentrated by evaporation and further purification was performed using reverse-phase high-performance liquid chromatography (RP-HPLC; Shimadzu, Japan). The column used Shim-Pack C_18_ (20 × 250 mm) and the mobile phases were distilled water and acetonitrile (ACN), containing 0.05% trifluoroacetic acid (TFA). A linear gradient of 30–46% over 25 min was used with a flow rate of 10 ml/min. The purified substances were lyophilized, and their molecular masses were identified by liquid chromatography electrospray ionization mass spectrometry (LC-ESI-MS; API2000, AB SCIEX, United States), in positive mode.

### *In vitro* Antibacterial Assay

The antibacterial assays of the culture filtrate of JCK-5075, crude substances (80% ethanol crude of Diaion HP-20), and PGPs, were conducted in a 96-well plate for microorganisms as previously reported, using the broth dilution method (Vu et al., 2017). Two-fold serial dilutions were performed with 10% of the culture supernatant, 100 μg ml^–1^ of crude substances, and 32 μg ml^–1^ of PGPs. Bacterial suspension (10^5^ CFU/ml) was added to each well and the final volume of the solution in each well was 100 μl. Streptomycin sulfate and oxolinic acid were chosen as the positive controls. TSB was used as an untreated control for the culture filtrate and 1% methanol for crude substances and PGPs. The plates were incubated at 28 or 30°C for 1–2 days and the growth of the pathogenic bacteria were measured by optical density (OD) at 600 nm by Microplate Reader (iMark, Bio-Rad). Each run of the experiment contained three replicates, and the entire experiment was run in entirely twice. Minimum inhibitory concentration (MIC) values were at the lowest culture filtrate concentration and the inhibition rate was calculated as follows:

(1) $$\begin{array}{c} \mathrm{Inhibition rate}\;(\%)\\ =\frac{\mathrm{OD of untreated control}-\mathrm{OD of treated}}{\mathrm{OD of untreated control}}\\ \;\times 100\% \end{array}$$

### *In vivo* Antibacterial Assays

#### Preparation of the Samples

The biocontrol efficacy of the fermentation broth of the *P. elgii* JCK-5075 was evaluated against Kimchi cabbage soft rot, tomato bacterial wilt, and red pepper bacterial leaf spot. In addition, PGP-A and PGP-C were tested against Kimchi cabbage soft rot and tomato bacterial wilt, respectively. The fermentation broth of *P. elgii* JCK-5075 was diluted with distilled water at 5, 10, and 20-fold, and then Tween 20 was added to each dilution at a concentration of 250 μg/ml. PGP-A and C were dissolved in methanol and then diluted with distilled water at concentrations of 200, 100, and 50 μg/ml, followed by the addition of Tween 20 (250 μg/ml). The final concentrations of the methanol in the chemical solutions were 5%. A commercial bactericide, Ilpum (Oxolinic acid 20% WP; Dongbang Agro, South Korea), and Tween 20 solution (250 μg/ml) either with or without methanol (5%), were used as the positive and negative controls, respectively. Ilpum was diluted 1000-fold, as recommended by the manufacturer.

#### Kimchi Cabbage Soft Rot

The disease control efficacy of the fermentation broth of *P. elgii* JCK-5075 and the purified PGP-A against Kimchi cabbage soft rot was performed on the sixth-leaf stage of the Kimchi cabbage plants. Out of the four PGPs, PGP-A showed the strongest activity against *P. carotovorum* subsp. *carotovorum* with a MIC value of 16 μg/ml (Table 2). Therefore, PGP-A was chosen for an *in vivo* assay against bacterial soft rot of the Kimchi cabbage caused by *P. carotovorum* subsp. *carotovorum*. The plants were treated with 20 ml of the fermentation broth or PGP-A solutions by soil drench. After 24 h, the treated plants were inoculated with 20 ml of the *P. carotovorum* subsp. *carotovorum* cell suspension (10^7^ CFU/ml, containing 10 mM MgCl_2_) by soil drench (Shrestha et al., 2009). The inoculated plants were incubated in the dark for 24 h at 30 ± 2°C and 100% RH, then kept in an incubation room with 12 h of daylight per day for 8 days. The disease severity (DS) was recorded on a scale ranging from 0 to 5 (Champoiseau et al., 2006). Where 0 = no symptom; 1 = one or two pencil-line streaks, 2 = more than two pencil-line streaks, 3 = leaf chlorosis or bleaching, 4 = leaf necrosis, and 5 = death of the plant.

#### Tomato Bacterial Wilt

The protective activity of the fermentation broth of the *P. elgii* JCK-5075 and the purified PGP-C against the tomato bacterial wilt was evaluated on the fourth-leaf stage tomato plants. *R. solanacearum* in the *in vitro* bioassay was sensitive to all four PGPs at 32 μg/ml, and the limited amounts of PGPs were obtained in this study. This allowed us to evaluate the biocontrol efficacy of the PGP-C alone against the tomato bacterial wilt in the pot experiments. The tomato plants were treated with 20 ml of the fermentation broth or PGP-C solutions by soil drench. After 24 h of treatment, the treated tomato plants were inoculated with 20 ml of the *R. solanacearum* cell suspension (10^8^ CFU/ml, 10 mM MgCl_2_) by soil drench. The inoculated plants were kept in the dark for 24 h at 30 ± 2°C, and then transferred to an incubation room at 30 ± 2°C, with 75% RH for 10 days, with 12 h of daylight per day. The DS on the tomato plants was assessed on a scale of 0–4 as follows (Vu et al., 2017). 0 = no leaf symptoms, 1 = one leaf wilted, 2 = two or three leaves wilted, 3 = more than four leaves wilted, and 4 = a dead plant.

#### Red Pepper Bacterial Leaf Spot

The disease control efficacy of the *P. elgii* JCK-5075 fermentation broth against red pepper bacterial leaf spot was carried out using sixth-leaf stage plants. The 5 ml aliquots of each sample were applied onto the red pepper plants and then the treated plants were inoculated with 5 ml of the *X. euvesicatoria* cell suspension (10^7^ CFU/ml) 24 h after the treatment by foliar spray. The inoculated plants were incubated in the dark at 25 ± 2°C and 100% RH for 24 h, they were then kept at 25 ± 2°C and 100% RH, for 16 days in a plastic cover with 12 h of daylight per day. The DS was recorded on a scale of 0–7 as follows (Abbasi et al., 2002). 0 = no symptom, 1 = symptomless, 2 = a few necrotic spots on a few leaflets, 3 = a few necrotic spots on many leaflets, 4 = many spots with coalescence on few leaf, 5 = many spots with coalescence on many leaflets, 6 = severe disease, and leaf defoliation, and 7 = a dead plant.

#### Calculation of the Control Value

The *in vivo* experiments were carried out with three replications and the entire experiment was repeated twice in entirely. The control value was calculated according to the following formula:

(2) $$Controlvalue\left(\%\right)=\frac{DSofcontrol-DSoftreatment}{DSofuntreatedcontrol}\times 100$$

### Calcein Leakage Assay

A calcein leakage assay was performed according to established protocols (Kim et al., 2018). Briefly, lipid mixtures consisting of 7:3 weight ratios of the phosphatidylcholine (POPC) and phosphatidylglycerol (POPG) were prepared from stock lipid solution in 100% methanol. The organic solvents were evaporated, and the resulting lipid film was lyophilized for 1 day. The dried lipid film was resuspended in 1 ml of dye buffer solution (70 mM calcein, 10 mM Tris–HCl, 0.1 mM EDTA, 150 mM NaCl, pH 7.4). Liposomes were prepared through 10 freeze-thaw cycles in liquid nitrogen, followed by an incubation in a 50°C water bath. Then, the suspensions were extruded through 100 nm pore-polycarbonate membranes 20 times. After extrusion, the calcein-entrapped large unilamellar vesicles (LUVs) were separated using gel filtration through a Sephadex G50 column and eluted with a buffer solution (10 mM Tris–HCl, 0.1 mM EDTA, 150 mM NaCl, pH 7.4). Calcein leakage from the LUVs was measured at 20°C by monitoring the fluorescence intensity at an excitation wavelength of 490 nm and an emission wavelength of 520 nm on a spectrophotometer (Cary 5000 UV-Vis-NIR). The peptide and lipid molar ratios ranged from 1:1 to 1:128. The liposome without the peptide was used as a negative control, and the Triton X-100 was used as a positive control. Each run of the experiment contained three replicates, and the entire experiment was run in entirely twice. The percentage of the calcein leakage was calculated according to the following formula:

(3) $$\begin{array}{c} \mathrm{Calcein leakage}\;(\%)\\ =\frac{\begin{array}{c} \mathrm{Fluoresence intensity of peptide treated}\\ -\mathrm{Fluoresence intensity without peptide} \end{array}}{\begin{array}{c} \mathrm{Fluorescent intensit y with Triton X}-100\\ -\mathrm{Fluorescence intensity without the peptide} \end{array}}\times 100\% \end{array}$$

### Cytoplasmic Membrane Depolarization Assay

Cytoplasmic membrane depolarization activity of the PGP-C peptide was determined using a potential sensitive dye, DiSC_3_-(5), as previously described (Wu and Hancock, 1999; Papo and Shai, 2005; Kim et al., 2018). Briefly, *X. oryzae* pv. *oryzae* and *R. solanacearum* were grown at 30°C to mid-log phase (OD_600 nm_ = 0.7) and were harvested by centrifugation. Cells were washed 3 times with buffer solution (5 mM HEPES, 20 mM glucose, pH 7.4) and gently resuspended to an OD_600 nm_ of 0.15 in the same buffer solution. Then, the cell suspension was incubated with 2 μM of DiSC_3_-(5) at room temperature for 1 h. DiSC_3_-(5) was used with 2 mM stock in dimethylsulfoxide (DMSO). Membrane depolarization was monitored using a F-4500 FL fluorescence spectrophotometer (Hitachi, Japan), with filter wavelengths of 620 and 670 nm for excitation and emission, respectively. The peptide was treated after 100 s and the peptide-free cells were used as a negative control.

### SYTOX Green Uptake Assay

The effect of the PGP-C peptide on the bacterial membrane permeabilization was evaluated using a SYTOX Green uptake assay, as previously described (Roth et al., 1997). Briefly, *X. oryzae* pv. *oryzae* and *R. solanacearum* were grown at 30°C to a mid-log phase (OD_600 nm_ = 0.7), they were then harvested and washed three times with 1 × PBS buffer (pH 7.4). The cells were gently resuspended to an OD_600 nm_ of 0.15 in the same buffer solution. Then, the cell suspension was incubated with 0.1 μM of SYTOX Green at room temperature for 16 h in the dark. SYTOX Green was used with 5 mM stock in DMSO. The uptake of the SYTOX Green was monitored using a F-4500 FL fluorescence spectrophotometer (Hitachi, Japan), with filter wavelengths of 480 and 520 nm for excitation and emissions, respectively. The peptide was treated after 100 s and the peptide-free cells were used as a negative control.

### Statistical Analysis

The *in vivo* data were analyzed for homogeneity of variances using the SPSS statistical analysis software (version 23 software; IBM, Armonk, NY, United States). Data are expressed as means ± standard deviations of the replicates and were analyzed by one-way-analysis of variance (ANOVA) and the statistical differences among the treatments were determined according to Duncan’s multiple-range test (*p* < 0.05), among the means using SPSS statistical analysis software.

## Results

### Antagonistic Activity of the *P. elgii* JCK-5075 Against Plant Pathogenic Bacteria

The JCK-5075 strain was identified as *P. elgii* with accession number MT176528 by phylogenetic analysis (Supplementary Figure S1). Fermentation filtrate of the *P. elgii* JCK-5075 exhibited a range of strong antibacterial effects against the 14 plant pathogenic bacteria, with MIC values of 0.63–10% (Table 1). Among the 14 bacteria tested, *X. arboricola* pv. *pruni* was the most sensitive to the fermentation filtrate and ethanol crude substances of *P. elgii* JCK-5075, with MIC values of 0.31 and 3.13 μg/ml, respectively, followed by *A. tumefaciens*, *X. euvesicatoria*, *X. axonopodis* pv. *citri*, and *X. oryzae* pv. *oryzae* with MIC values of 0.63 and 6.25 μg/ml. *A. avenae* subsp. *cattleyae*, *A. konjaci*, and *P. carotovorum* subsp. *carotovorum* were also highly sensitive, with MIC values ranging from 1.25 to 2.5% for the fermentation filtrate, and from 12.5 to 25 μg/ml for the crude substances. In addition, the growth of five of the pathogenic bacteria including, *B. glumae*, *C. michiganensis* subsp. *michiganensis*, *P. syringae* pv. *actinidiae*, *P. syringae* pv. *lachrymans*, and *R. solanacearum*, were relatively resistant to the antibacterial metabolites of JCK-5075 with MIC values of 10% and 100 μg/ml for the fermentation filtrate and the crude substances, respectively. Oxolinic acid, a synthetic quinolone antibiotic, inhibited the growth of all the pathogenic bacteria tested with MIC values of 0.05–3.13 μg/ml. Out of the 14 plant pathogenic bacteria, four strains, *A. avenae* subsp. *cattleyae*, *P. chrysanthemi*, *A. tumefaciens*, *X. euvesicatoria*, were resistant to streptomycin sulfate but they were sensitive to the fermentation filtrate and crude substances of *P. elgii* JCK-5075 (Table 1).

**TABLE 1:** Minimum inhibitory concentration (MIC) of the fermentation filtrate and the ethanol crude substances of *Paenibacillus elgii* JCK-5075 against 14 plant pathogenic bacteria.

Plant pathogenic bacteria	MIC			
	*P. elgii* JCK-5075		Streptomycin sulfate (μg/ml)	Oxolinic acid (μg/ml)
	Fermentation filtrate (%)	Crude substances (μg/ml)		
*A. avenae* subsp. *cattleyae*	1.25	12.50	>200.00	0.05
*A. konjaci*	2.50	25.00	6.25	0.78
*A. tumefaciens*	0.63	6.25	200.00	0.78
*B. glumae*	10.00	100.00	12.50	0.10
*C. michiganensis* subsp. *michiganensis*	10.00	100.00	12.50	0.10
*P. carotovorum* subsp. *carotovorum*	2.50	25.00	12.50	1.56
*P. chrysanthemi*	5.00	50.00	>200	0.10
*P. syringae* pv. *actinidiae*	10.00	100.00	12.50	3.13
*P. syringae* pv. *lachrymans*	10.00	100.00	12.50	1.56
*R. solanacearum*	10.00	100.00	6.25	0.19
*X. euvesicatoria*	0.63	6.25	50.00	0.10
*X. arboricola* pv. *pruni*	0.31	3.13	12.50	0.39
*X. axonopodis* pv. *citri*	0.63	6.25	50.00	0.78
*X. oryzae* pv. *oryzae*	0.63	6.25	6.25	0.78

### Purification of the PGPs From the Fermentation Supernatant of *Paenibacillus elgii* JCK-5075

Purification of the PGPs from the culture broth was performed on a Diaion HP-20 column chromatography and preparative RP-HPLC. Of the eluted fractions, the 80% ethanol fraction contained PGPs including [M + H]^+^ ion peaks at *m/z* 1073.3, 1011.6, 1087.3, and 1087.2 Da, which corresponded to the masses of the PGP-A, B, C, and D, respectively. We further purified using preparative RP-HPLC to obtain pure PGPs. The purity was confirmed using LC-ESI-MS spectrometry (Supplementary Figure S2). The following amounts of the PGPs were obtained from the 1 L cultivation: 3.2 mg of PGP-A, 1.1 mg of PGP-B, 5.1 mg of PGP-C, and 1.4 mg of PGP-D.

### Antibacterial Activity of PGPs Against Plant Pathogenic Bacteria

The four PGPs isolated from the fermentation broth of the *P. elgii* JCK-5075, exhibited strong antibacterial activity against a wide spectrum of plant pathogenic bacteria (Table 2). Among the four metabolites, PGP-A, C, and D presented similar antibacterial activity, while PGP-B was the least active. *Xanthomonas* species including *X. euvesicatoria*, *X. arboricola* pv. *pruni*, *X. axonopodis* pv. *citri*, and *X. oryzae* pv. *oryzae* were the most sensitive, with all the PGPs, with MIC values from 2 to 16 μg/ml. The *B. glumae, C. michiganensis* subsp. *michiganensis*, *P. carotovorum* subsp. *carotovorum, P. chrysanthemi, P. syringae* pv. *actinidiae*, *P. syringae* pv. *Lachrymans*, and *R. solanacearum* were relatively insensitive to the PGPs.

**TABLE 2:** The minimum inhibitory concentration (MIC) of the pelgipeptins (PGPs) isolated from *Paenibacillus elgii* JCK-5075 against 14 plant pathogenic bacteria.

Plant pathogenic bacteria	MIC (μg/ml)			
	PGP-A	PGP-B	PGP-C	PGP-D
*A. avenae* subsp. *cattleyae*	8	32	8	8
*A. konjaci*	16	32	8	16
*A. tumefaciens*	8	16	4	4
*B. glumae*	>32	>32	32	32
*C. michiganensis* subsp. *michiganensis*	16	>32	32	32
*P. carotovorum* subsp. *carotovorum*	16	>32	32	32
*P. chrysanthemi*	16	32	32	32
*P. syringae* pv. *actinidiae*	32	>32	32	32
*P. syringae* pv. *lachrymans*	32	>32	32	32
*R. solanacearum*	32	>32	32	32
*X. euvesicatoria*	4	16	2	4
*X. arboricola* pv. *pruni*	2	8	2	2
*X. citri*	4	16	4	4
*X. oryzae* pv. *oryzae*	4	16	4	4

### Biocontrol Efficacy of the JCK-5075 and PGPs in Controlling Bacterial Plant Diseases

**FIGURE 1:**
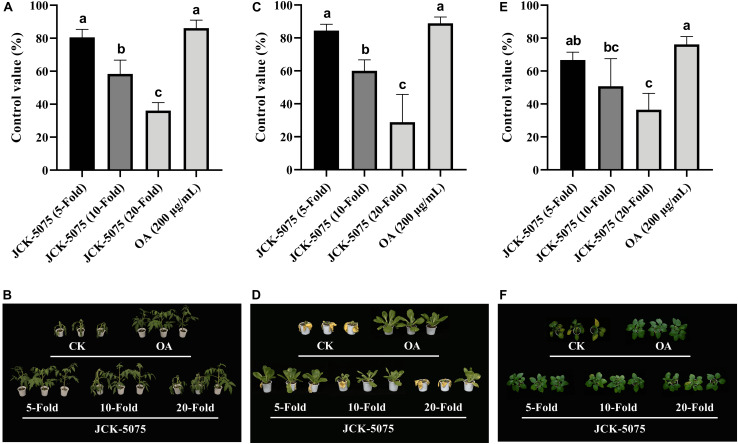
The biocontrol efficacy of the fermentation broth of *Paenibacillus elgii* JCK-5075 on tomato bacterial wilt **(A,B)**, Kimchi cabbage bacterial soft rot **(C,D)**, and red pepper bacterial leaf spot diseases **(E,F)**. Each value represents the mean ± standard deviation (*n* = 3) of two runs, with three replicates each. Means followed by the same letters above the bars are not significantly different (*p* < 0.05) in a Duncan’s multiple range test. JCK-5075 ( 5-, 10-, and 20-fold) are 5-, 10-, and 20-fold dilutions of the fermentation broth of JCK-5075; OA, oxolinic acid (200 μg/ml).

The fermentation broth of the *P. elgii* JCK-5075, suppressed the development of the tomato bacterial wilt, Kimchi cabbage soft rot, and red pepper bacterial leaf spot, in a dose-dependent manner (Figure 1). The control values of the fermentation broth of the *P. elgii* JCK-5075 against the tomato bacterial wilt were 81, 58, and 36%, at 5-, 10-, and 20-fold dilutions, respectively. In comparison, the commercial antibiotic Ilpum displayed a disease control efficacy of 86%, comparable to that of the 5-fold dilution of the fermentation broth of the JCK-5075. The fermentation broth of the JCK-5075 also reduced the development of the Kimchi cabbage soft rot, showing control values of 84, 60, and 29%, for the 5-, 10-, and 20-fold dilutions, respectively. The control value of the 5-fold dilution of the fermentation broth was not significantly different from that of Ilpum (89%). The control values of the fermentation broth of JCK-5075 against the red pepper bacterial leaf spot were 67, 51, and 37%, for the 5-, 10-, and 20-fold dilutions, respectively. The disease control efficacy (76%) of the Ilpum against the red pepper bacterial leaf spot was not significantly different from that of the 5-fold dilution of the fermentation broth of the JCK-5075.

**FIGURE 2:**
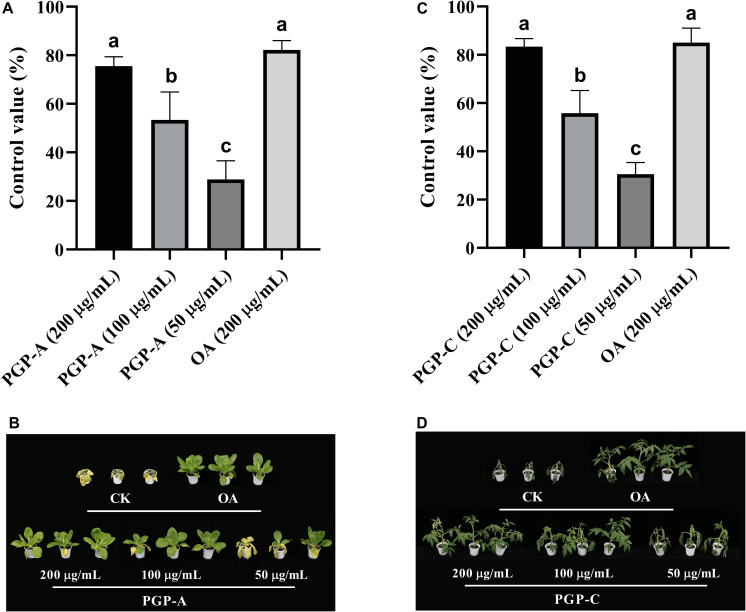
The disease-control efficacy of pelgipeptin (PGP)-A and C on Kimchi cabbage bacterial soft rot **(A,B)** and tomato bacterial wilt **(C,D)**, respectively. Each value represents the mean ± standard deviation (*n* = 3) of two runs, with three replicates each. Means followed by the same letters above the bars are not significantly different (*p* < 0.05) in a Duncan’s multiple range test. OA, oxolinic acid (200 μg/ml).

PGP-A also effectively suppressed the development of the Kimchi cabbage soft rot with control values of 76, 53, and 29% for 200, 100, and 50 μg/ml (Figure 2). The control value of the 200 μg/ml of the PGP-A was comparable to that of the oxolinic acid (200 μg/ml). On the other hand, PGP-C reduced the tomato bacterial wilt by 83, 56, and 31%, for the 200, 100, and 50 μg/ml, respectively. The disease control efficacy of the 200 μg/ml PGP-C was comparable to that of the oxolinic acid (200 μg/ml).

### Action Mechanisms of the PGP-C

**FIGURE 3:**
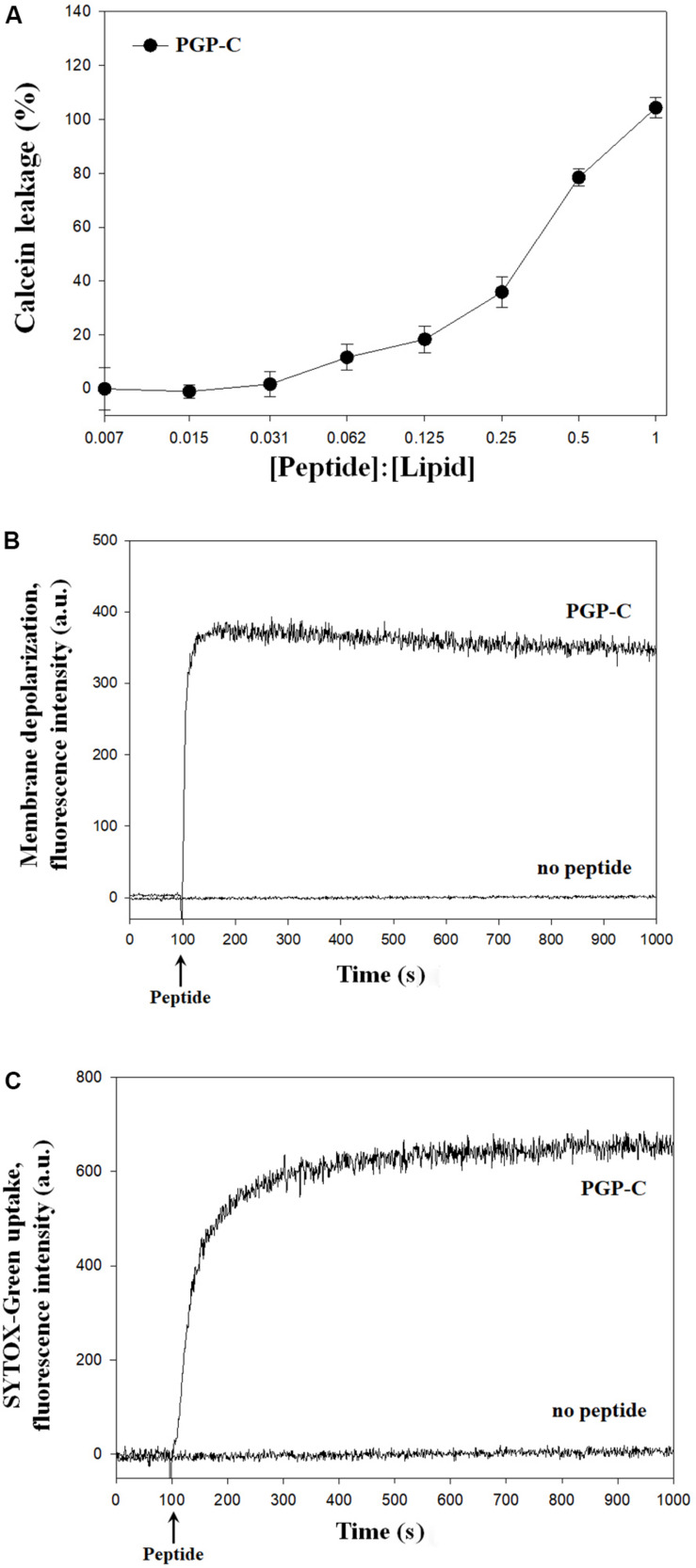
The mechanism studies of pelgipeptin (PGP)-C using calcein leakage **(A)**, membrane depolarization **(B)**, and SYTOX-Green uptake **(C)** experiments. For calcein leakage, PGP-C was added to calcein encapsulated large unilamellar vesicles (LUVs) at different peptide:lipid ratios. Each value represents the mean ± standard deviation (*n* = 3) of two runs, with three replicates each. The depolarization of the *X. oryzae* pv. *oryzae* cytoplasmic membrane was induced by the PGP-C peptide at 8 μg/ml concentration. The alterations of the cytoplasmic membrane of *X. oryzae* pv. *oryzae* by PGP-C (8 μg/ml) allowed the SYTOX Green probe to enter the cell and bind DNA, resulting in an increase of fluorescence.

The mechanisms of the antibacterial action of the PGP-C peptide were assessed by calcein leakage, membrane depolarization, and SYTOX-Green uptake. First, the calcein leakage assay was performed by mixing the PGP-C with 70 mM calcein encapsulated LUVs, at peptide:lipid molar ratios ranging from 1:128 to 1:1. Three measurements were averaged and plotted against the peptide:lipid ratios. PGP-C peptide gradually increased the calcein dye leakage by increasing the peptide ratio (Figure 3A). At a very low peptide:lipid ratio of 0.031 or less, the leakage did not exceed 10%, but after that the leakage gradually increased to reach 100% at 1.0 ratio. For the membrane depolarization and SYTOX-Green uptake experiments, we used a *Xanthomonas* strain (*X. oryzae* pv. *oryzae*), which was more sensitive to PGP peptides than other strains (Table 2). The potentiometric probe DiSC_3_-(5) is a carbocyanine with a short (C_3_) alkyl tail. This cationic dye accumulates on hyperpolarized membranes and is translocated into the lipid bilayer. When the cytoplasmic membrane is damaged, the membrane potential is dissipated and the DiSC_3_-(5) dye is released, causing an increase in fluoresce. The PGP-C with 2 × MIC (8 μg/ml) induced the complete depolarization of the cytoplasmic membrane within a few seconds (Figure 3B). We also applied a SYTOX-Green uptake assay to examine the ability of the PGP-C peptide to affect the bacterial membrane permeability, by monitoring the intracellular influx of the SYTOX-Green. The SYTOX-Green is a bright and high-affinity nucleic acid stain that easily penetrates the cells with damaged plasma membranes but does not cross the membranes of the living cells. The intensity of the fluorescence was immediately increased by the addition of the PGP-C (2 × MIC) (Figure 3C). Without the peptide, the fluorescence intensities in both the membrane depolarization and SYTOX-Green uptake experiments were not changed (Figures 3B,C), suggesting that the PGP-C specifically induced the increasing fluorescence by disrupting the membrane integrity.

## Discussion

Many bacterial strains display their antagonistic activity both *in vitro* and *in vivo*, but only a few are successful for the control of plant diseases under field conditions (Shoda, 2000; Lugtenberg and Kamilova, 2009; Xu and Kim, 2014; Tsuda et al., 2016). The antimicrobial compounds produced by bacteria can be useful as biocontrol measures in agriculture, as demonstrated in several previous studies (Chung et al., 2000; Beatty and Jensen, 2002; Antonopoulos et al., 2008; Allard et al., 2014; Grady et al., 2016). Like the *Bacillus* spp., members of the *Paenibacillus* species produce a wide array of antimicrobial compounds, to compete with other microorganisms (Chung et al., 2000; Shaheen et al., 2011; Kai et al., 2013). Bacteria belonging to the *Paenibacillus* species were isolated from a variety of different environments, and their biological efficacy was different even within the same species; among the 25 strains of *P. polymyxa* isolated from ginseng roots, 15 strains showed strong activity against *Phytophthora capsici*, while 10 strains showed weak or no antimicrobial effects (Kim et al., 2009). In this study, *P. elgii* JCK-5075 presented strong antibacterial activity against various plant pathogenic bacteria. The fermentation filtrate of *P. elgii* JCK-5075 inhibited the growth of all 14 bacteria that were tested, with MIC values that ranged from 0.31 to 10%. The crude substances (80% ethanolic fraction of Diaion HP-20) also displayed antibacterial activity against all pathogenic bacteria, having a MIC range of 3.13–100 μg/ml (Table 1). Several studies reported on the disease control efficacy of *Paenibacillus* species against soil-borne diseases (Antonopoulos et al., 2008; Khan et al., 2008; Algam et al., 2010; Xu and Kim, 2014). To date, the information about the control of bacterial plant diseases using *P. elgii* species has been unavailable. Our results indicated the potential of *P. elgii* JCK-5075 for controlling plant diseases caused by pathogenic bacteria.

The well-known *P. elgii* strain may produce various antimicrobial compounds including PGPs, protocatechuic acid, and methyl 2,3-dihydroxybenzoate (Wu et al., 2010; Ding et al., 2011; Nguyen et al., 2015; Lee et al., 2017; Kim et al., 2018). PGPs, a class of cyclic lipopeptides, were first isolated from the fermentation broth of *P. elgii* B69 and displayed strong activity against human pathogenic bacteria in the range of 0.78–100 μg/ml, and PGP-B was the most active (Wu et al., 2010; Ding et al., 2011). PGP-E isolated by *P. elgii* BC34-6 also showed strong antibacterial activity, including methicillin-resistant *Staphylococcus aureus* with MICs of 2–8 μg/ml (Kim et al., 2018). In another report, PGP-D that was isolated from *P. elgii* AC13, showed the strongest antibacterial activity among the four types of PGP (Costa et al., 2019). In this study, 4 PGPs, namely A, B, C, and D, were isolated from the fermentation supernatant of *P. elgii* JCK-5075, showed strong *in vitro* antibacterial activity against 14 plant pathogenic bacteria, with MIC values of 2–32 μg/ml. Even though the antibacterial activity of the four chemicals varied depending on the test pathogens, PGP-A and C displayed the strongest activity, followed by PGP-D and B. Our data differ from the results of previous reports (Wu et al., 2010; Ding et al., 2011; Costa et al., 2019). This may be due to the different target bacteria, as the sensitivity of the bacteria to chemicals varies with the strains, even within one species.

The biological efficacy of the *P. elgii* JCK-5075 was confirmed herein, to control bacterial plant diseases in pot experiments. The fermentation broth of *P. elgii* JCK-5075, effectively controlled tomato bacterial wilt, Kimchi cabbage soft rot, and red pepper bacterial leaf spot. *R. solanacearum*, a causal agent of bacterial wilt in various crops, causes vascular disease, and is one of the most destructive soil-borne pathogens (Mansfield et al., 2012). Physical, chemical, and biological methods were applied to control this disease, and the biological methods have been of interest to researchers for decades. Algam et al. (2010) reported that the disease incidence of the tomato bacterial wilt was reduced by 82% when *P. polymyxa* MB02-1007 was applied (Algam et al., 2010). Similar results were obtained from the treatment with the fermentation broth of *P. elgii* JCK-5075 in our study, having a control value of 80.55% at a 5-fold dilution.

*Pectobacterium carotovorum* is also one of the most destructive soil-borne plant pathogens and causes soft rot to many economically important crops (Perombelon and Kelman, 1980; Charkowski, 2018). Several reports have previously presented the potential of *Paenibacillus* species as biological controls against the soft rot of Kimchi cabbage (Vanneste et al., 1998; Kyeremeh et al., 2000; Li et al., 2014; Tsuda et al., 2016). Shrestha et al. (2009) reported the biocontrol efficacy of *Paenibacillus* KPB3 against Kimchi cabbage soft rot, with control values of 58 and 62% in the greenhouse and field tests, respectively (Shrestha et al., 2009). In this study, the 5-fold dilution of the fermentation broth of *P. elgii* JCK-5075 displayed a control value of 84.45% in pot experiments.

Bacterial leaf spot disease occurs on red pepper and tomato worldwide. Bacterial leaf spot is a common disease on red pepper plants in Korea and the causal pathogen was identified as *X. euvesicatoria*. Repeated use of streptomycin and copper compounds has resulted in the spread of resistant strains (Marco and Stall, 1983; Araújo et al., 2012). The strain of *X. euvesicatoria* used in this study is resistant to streptomycin sulfate with a MIC value of 50 μg/ml. The fermentation broth of *P. elgii* JCK-5075, at a 5-fold dilution, displayed a control value of 67%, which was not significantly different to that of oxolinic acid. *P. macerans* was reported to control leaf spot disease on tomato, having a biocontrol efficacy of higher than 50% (Lanna Filho et al., 2010). None of the plants treated with the 5-fold dilutions of the fermentation broth of the *P. elgii* JCK-5075, showed any phytotoxic symptoms (Sarwar et al., 2018). To the best of our knowledge, this is the first report on the efficacy of *P. elgii* to control plant bacterial diseases.

*Paenibacillus* species were reported to protect crops from pathogen infections using mechanisms such as induced systemic resistance (Khan et al., 2008; Lee et al., 2012; Sang et al., 2014) or the production of antimicrobial substances (Hong and Meng, 2003; Shaheen et al., 2011; Huang et al., 2013). Bacterial lipopeptides have a wide range antimicrobial activities against pathogenic microorganisms and even some resistant strains, in both medicine and agriculture (Cochrane and Vederas, 2016). The lipopeptides produced by *Bacillus amyloliquefaciens* strain FJAT-2349, a mixture of iturin A, fengycin, and surfactin, could effectively control tomato bacterial wilt, with a biocontrol efficacy of 97.6% (Chen et al., 2019). Rice bakanae disease was reduced up to 80% with surfactin A purified from *Bacillus* strains AH-100 and NH217. In our study, PGPs (A and C), at 200 μg/ml, significantly reduced the disease severity in Kimchi cabbage soft rot and tomato bacterial wilt, with control values of 76 and 83%, respectively. Since their first isolations from the *Paenibacillus* spp., the PGPs have not been applied commercially.

Based on the membrane depolarization and SYTOX-Green uptake assay, PGP-C strongly affected the bacterial membranes of the *X. oryzae* pv. *oryzae* at concentrations of 8 μg/ml. However, at the same concentration, PGP-C caused a similar but substantially slower depolarization on the *R. solanacearum* cytoplasmic membrane (Supplementary Figure S3A). Moreover, the SYTOX-Green uptake experiment revealed that the PGP-C peptide cannot induce membrane permeabilization of *R. solanacearum* at the same concentration (Supplementary Figure S3B). The MIC of PGP-C, against *R. solanacearum* is much higher than that of *X. oryzae* pv. *oryzae* (32 vs. 4 μg/ml), indicating that the PGP-C is much more active toward *X. oryzae* pv. *oryzae* than *R. solanacearum*. PGP-C is not able to kill *R. solanacearum* at 8 μg/ml concentrations. These results suggest that the antibacterial activity of PGP-C is directly correlated with the membrane damage mechanisms by pore-formation or structural disruption of the bacterial membranes.

## Conclusion

In this study, four members of the PGP family that were isolated from the fermentation supernatant of *P. elgii* JCK-5075, were found to have strong and broad-spectrum antibacterial activity against plant pathogenic bacteria. PGP-A and C also effectively controlled Kimchi cabbage soft rot and tomato bacterial wilt, respectively, and their disease control efficacy was comparable to that of a commercial bactericide, oxolinic acid. Additionally, PGP-C was found to impact on bacterial cell growth via membrane-active mechanisms. These results indicated that *P. elgii* JCK-5075 producing PGPs could be used as a biocontrol agent for the control of plant bacterial diseases. This is the first report on the *in vitro* and *in vivo* antibacterial activity of PGPs against bacterial plant pathogens.

## Data Availability Statement

The raw data supporting the conclusions of this article will be made available by the authors, without undue reservation, to any qualified researcher.

## Author Contributions

J-CK and CL designed the study. KL and JK designed and performed the experiments and analyzed the data. NY and BK performed the RNA isolation and identified species. J-CK, CL, KL, and JK wrote and revised the manuscript. All authors read and approved the final version of this manuscript.

## Conflict of Interest

The authors declare that the research was conducted in the absence of any commercial or financial relationships that could be construed as a potential conflict of interest. The authors also declare that they will apply for one patent using the results of this study.
