# Supplementary material for: Biological Control of Tomato Bacterial Wilt, Kimchi Cabbage Soft Rot, and Red Pepper Bacterial Leaf Spot Using Paenibacillus elgii JCK-5075
Source: Front Plant Sci. 2020 Jul 1;11:775. doi: 10.3389/fpls.2020.00775 (PMC7340725; doi:10.3389/fpls.2020.00775)
Supplement: Supplementary file 1 [file Data_Sheet_1.docx]

**Supporting Information**

**
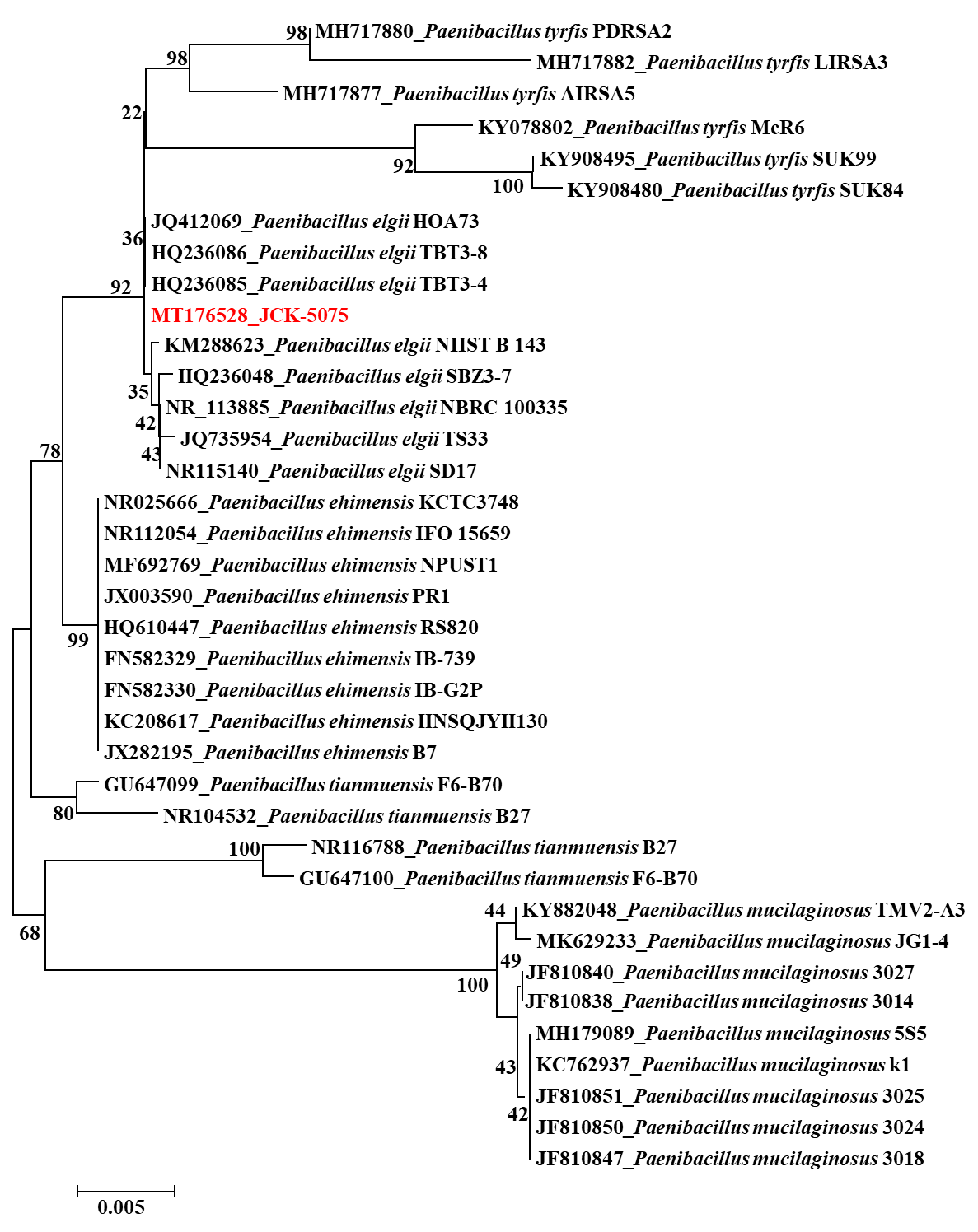
**

**FIGURE S1 |** Neighbor-joining phylogenetic tree of JCK-5075 based on 16S rRNA sequences analysis. The number at nodes indicates the level of bootstrap support (%) based on 1000 replicates dataset. The scale bar at the bottom indicates genetic distance unit.

**
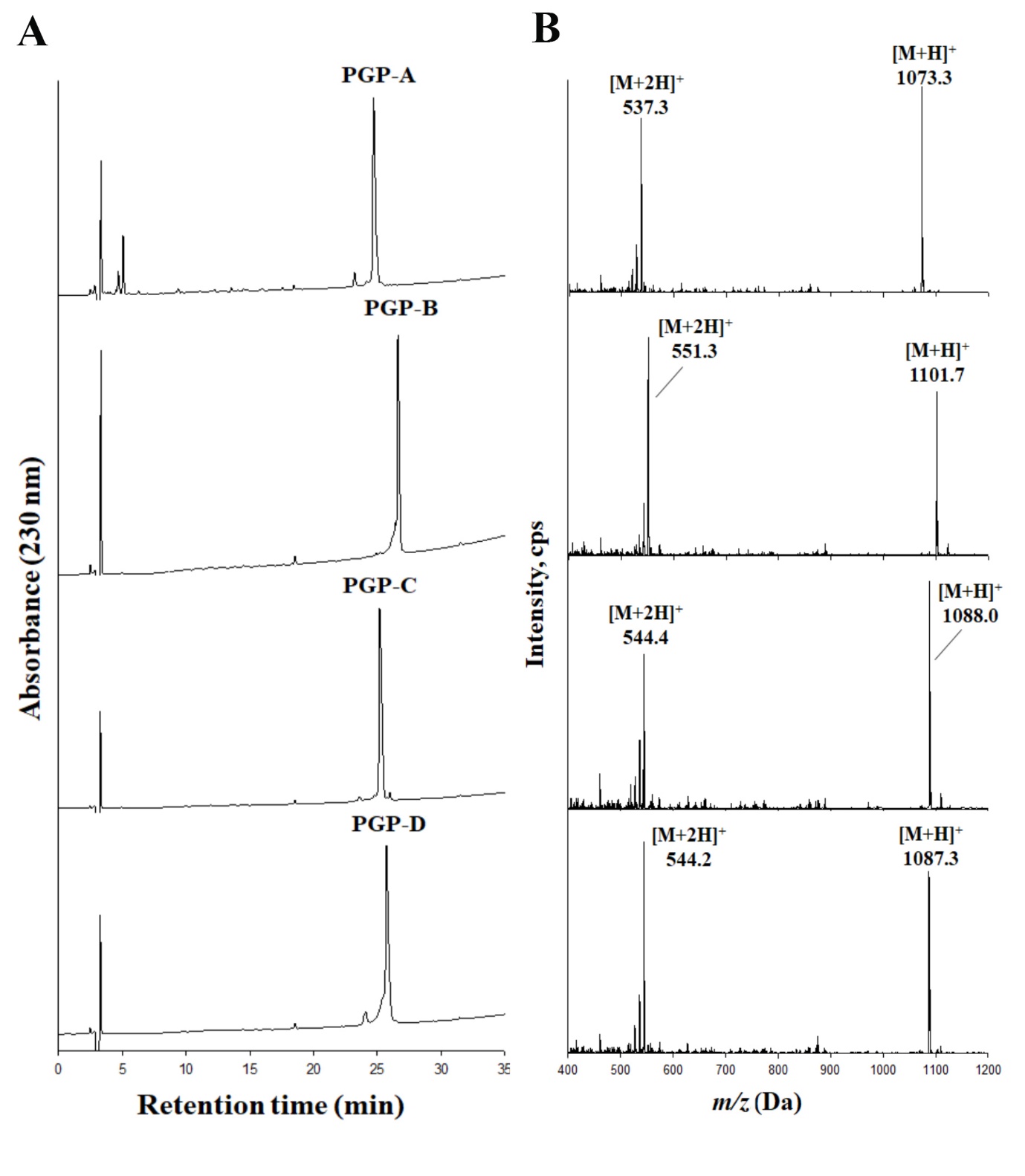
**

**FIGURE S2 |** Liquid chromatography electrospray ionization mass spectrometry (LC-ESI-MS) analysis of the purified pelgipeptins (PGPs). Reverse-phase high performance liquid chromatography (RP-HPLC) chromatogram (**A**), ESI-MS spectrum in positive mode (**B**).

**
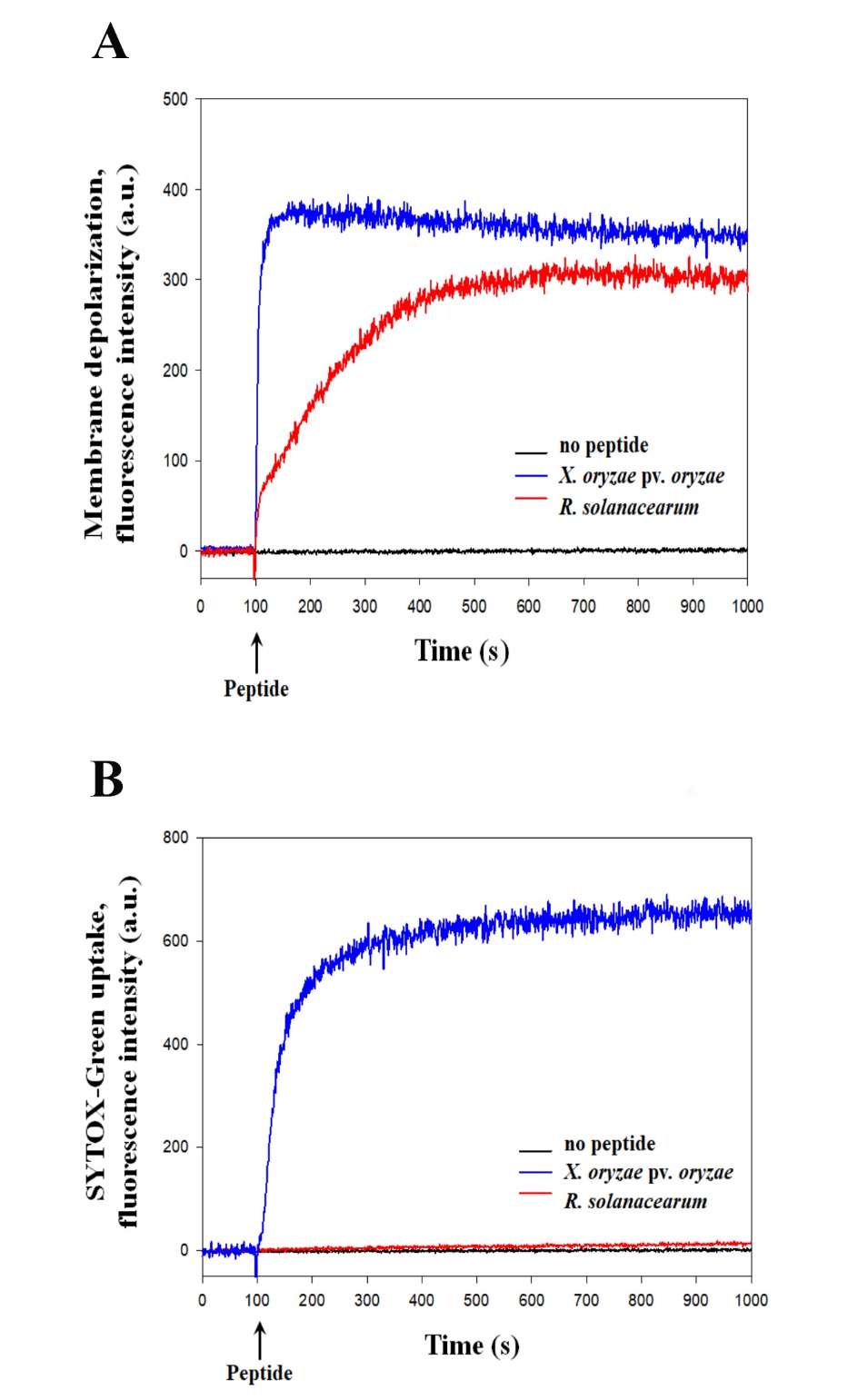
**

**FIGURE S3** **|** The comparison of the cell membrane depolarizations. The depolarization of the cytoplasmic membrane was induced by pepgipeptin-C (PGP-C) peptide at 8 µg/ml, that was determined using the membrane potential-sensitive fluorescent dye DiSC3-(5) (**A**). The comparison of cell membrane permeabilization caused by PGP-C peptide at 8 µg/ml (**B**).
